# Cost-effectiveness of adoption strategies for point of care HIV viral load monitoring in South Africa

**DOI:** 10.1016/j.eclinm.2020.100607

**Published:** 2020-11-04

**Authors:** Sarah J. Girdwood, Thomas Crompton, Monisha Sharma, Jienchi Dorward, Nigel Garrett, Paul K. Drain, Wendy Stevens, Brooke E. Nichols

**Affiliations:** a Health Economics and Epidemiology Research Office, Department of Internal Medicine, School of Clinical Medicine, Faculty of Health Sciences, University of the Witwatersrand, Johannesburg, South Africa; b Department of Viroscience, Erasmus Medical Centre, Rotterdam, the Netherlands; c Strategic Information Analytics, Right to Care, Johannesburg, South Africa; d Departments of Global Health, Medicine, and Epidemiology, University of Washington, Seattle, WA, USA; e Centre for the AIDS Programme of Research in Africa (CAPRISA), University of KwaZulu-Natal, Durban, South Africa; f Nuffield Department of Primary Care Health Sciences, University of Oxford, Oxford, United Kingdom; g Discipline of Public Health Medicine, School of Nursing and Public Health, University of KwaZulu-Natal, Durban, South Africa; h National Health Laboratory Service, Johannesburg, South Africa; i Department of Molecular Medicine and Haematology, Faculty of Health Sciences, University of the Witwatersrand, Johannesburg, South Africa; j Department of Global Health, Boston University School of Public Health, 801 Massachusetts Ave, Crosstown Center, 3rd floor, Boston, MA 02118, USA

**Keywords:** Viral load scale-up, Point of care, South Africa, Cost-effectiveness

## Abstract

**Background:**

Viral load (VL) testing is recommended for monitoring people on ART. The National Health Laboratory Service (NHLS) in South Africa conducts >5million laboratory-based VL tests but faces challenges with specimen integrity and results delivery. Point-of-care (POC) VL monitoring may improve VL suppression (VLS). We assessed the cost-effectiveness of different strategies for POC testing in South Africa.

**Methods:**

We developed a cost-outcome model utilizing NHLS data, including facility-level annual VL volumes, proportion with VLS, specimen rejection rates, turn-around-time, and the cost/test. We assessed the impact of adopting POC VL technology under 4 strategies: (1) status-quo; (2) targeted POC testing at facilities with high levels of viral failure; (3) targeted POC testing at low-performing facilities; (4) complete POC adoption. For each strategy, we determined the total cost, effectiveness (expected number of virally suppressed people) and incremental cost-effectiveness ratio (ICER) based on expected (>10%) VLS improvement.

**Findings:**

Existing laboratory-based VL testing costs $126 m annually and achieves 85.2% VLS. Strategy 2 was the most cost-effective approach, with 88.5% VLS and $40/additional person suppressed, compared to the status-quo. Should resources allow, complete POC adoption may be cost-effective (ICER: $136/additional person suppressed), requiring an additional $49 m annually and achieving 94.5% VLS. All other strategies were dominated in the incremental analysis.

**Interpretation:**

Assuming POC VL monitoring confers clinical benefits, the most cost-effective strategy for POC adoption in South Africa is a targeted approach with POC VL technologies placed at facilities with high level of viral failure.

**Funding:**

Funding support from the Bill & Melinda Gates Foundation.

**Research in Context**

**Evidence before this study**

We searched PubMed for studies published up to 15 July 2020 with the search terms “point-of-care” and “cost-effectiveness” and “viral load” with no language or date restrictions. Relevant studies have assessed POC diagnostic accuracy, costs, cost-effectiveness, and clinical outcomes relative to centralized testing. We did not find studies assessing POC testing performance in low throughput settings or analyses of the cost-effectiveness of POC VL testing relative to conventional centralized laboratory testing at scale; nor did we find studies providing decision-makers with information on how to incorporate POC instruments into a national viral load monitoring program such that instrument utilization and outcomes are optimized.

**Added value of this study**

To our knowledge, this is the first paper to assess the feasibility of different adoption strategies for POC viral load monitoring on a national scale. This analysis is novel in that it incorporates both costs and outcomes and uses facility-level data to match viral load demand to equipment capacity in order to minimize costs. This study can provide insight to resource-limited settings in implementing POC viral load adoption strategies.

**Implications of all the available evidence**

POC viral load testing has been demonstrated to improve the proportion of people virally suppressed on ART. Used strategically, POC can be an important complementary strategy to conventional centralized testing in a national viral load program and can improve access for harder to reach facilities/populations and/or improve outcomes.

Alt-text: Unlabelled box

## 1 Introduction

The World Health Organization (WHO) recommends viral load testing for monitoring persons living with HIV (PLHIV) on ART [1]. However, providing viral load monitoring to the millions of PLHIV in HIV care remains challenging in resource-limited settings. In South Africa, the National Health Laboratory Service (NHLS) is the largest diagnostic pathology service provider and provides laboratory and related public health services to over 80% of the population. In 2018, the NHLS operated a highly centralized national viral load network that conducted more than five million viral load tests at 16 laboratories. Despite this wide network, the system faces challenges regarding specimen integrity, and result delivery, which can cause delays in clinical decision making and timely adherence counseling provision [2]. Currently, only 69% of PLHIV on ART in South Africa receive viral load testing in accordance with the guideline-recommended schedule [3]. Timely viral load testing is the first essential step to detecting a person with an unsuppressed viral load and to implement the appropriate clinical action (adherence counseling and/or switch to second-line ART) [4]. Persons with long-term unsuppressed viral loads who do not receive appropriate clinical action are at risk for poorer health outcomes and/or onward HIV transmission [5]. Improving viral load testing coverage in accordance with national guidelines is critical to aid in South Africa's achievement of high levels of viral suppression to meet the last 95% of UNAIDs ambitious HIV targets.

Point of care (POC) viral load technologies can be used to achieve two (non-mutually exclusive) primary goals: (1) increasing viral load access (e.g. bringing viral load testing to remote areas or to patients who have not typically accessed viral load testing), and (2) improving patient outcomes by providing an immediately actionable test result and decreasing the amount of time patients spend virally unsuppressed. A recent trial conducted in South Africa found that POC viral load testing improved patient retention in care by 7·7% (85–92%) and viral suppression (<200 copies/ml) by 12·4% at 18 months post treatment initiation for patients on ART who accessed a viral load through POC (93% viral suppression) as compared to standard centralized testing (83% viral suppression) [6]. A study in Malawi found that near-POC viral load testing targeted at patients suspected of treatment failure or returning to care following a previously elevated viral load, was feasible and consistently enabled prompt clinical action [7]. Another study in Malawi found that POC viral load testing increased clinically indicated ART regimen switches (86% vs 67%) and reduced the time to switching (6.8 months vs 9.7 months) [8]. However, there is a lack of data about whether POC viral load testing adoption in a clinic-based setting is cost-effective relative to conventional centralized laboratory testing in resource-limited settings. Cost-effectiveness of POC viral load testing adoption strategies are likely to be influenced by a number of factors including instrument utilization, equipment cost, instrument placement, test accuracy in decentralized settings, and impact on patient clinical outcomes [9,10]. We sought to assess the cost-effectiveness of different adoption strategies for POC HIV viral load monitoring to improve patient outcomes in South Africa.

## 2 Methods

We developed a geospatial cost-outcome model utilizing existing aggregate data from NHLS in order to assess the cost per outcome for the entire system taking into account facility-level heterogeneity. We used data for all facilities in South Africa that send blood specimens to centralized NHLS laboratories for viral load testing for the 2018 calendar year, including annual viral load volumes; suppression rates (<1000 copies/ml); specimen rejection rates; turn-around time (TAT) measured from when the specimen is registered at the laboratory until the results are reviewed; and the cost per viral load test by testing platform. We considered different POC adoption strategies: integrating and leveraging ‘additional capacity’ currently available on instruments in-country; targeting POC test use for certain facilities or to certain patient populations within facilities; and/or prioritizing POC instrument placement at facilities that perform poorly in terms of specimen rejection and clinical action delays.

We included different instruments on the market that perform POC viral load tests; the Xpert® HIV-1 Viral Load (Cepheid, Sunnyvale, USA) (“Xpert”) and the newly available m-PIMA™ HIV-1/2 Viral Load (Abbott, USA) (m-PIMA)[11]. Whilst the NHLS currently uses 4, 16 or 80 module Xpert instruments for tuberculosis diagnosis, these instruments are located at laboratories and have not been used in a POC setting, nor have they been used for HIV viral load testing in South Africa. Abbott's m-PIMA™ offers the advantage of being better suited to operating in a true POC setting operated by lay healthcare workers, but is typically more costly than the Xpert (Table 2) and only comes in a single module instrument [11]. As such, we assessed the impact of the adoption of these two validated viral load POC technologies, the Xpert and m-PIMA, under four strategies (Table 1).

**Table 1:** Strategy facility selection criteria.

Strategies	Description	Selection criteria
1. Status quo	All viral load specimens are sent for centralized testing.	All facilities in South Africa that currently send viral load specimens to centralized laboratories
2. Unsuppressed targeted	Targeted POC for patients suspected of viral failure at facilities with low suppression rates/high numbers of unsuppressed viral loads	Facilities with at least one unsuppressed viral load expected daily or facilities with the lowest viral suppression rates (less than 80% – quintile 4 and 5) but at least 4 viral loads expected per day were selected for this strategy. Only primary healthcare facilities and district level hospitals were included.
3. Combination targeted	POC coverage at facilities with a combination of low suppression rates, and high rejection rates and TAT	Facilities were divided into quintiles according to their viral load specimen rejection rates, viral load suppression rates, and TAT. Facilities were scored based on their total quintile score, and sorted in descending order. The lowest performing facilities according to these indicators (low viral suppression, long TAT and high specimen rejection rates) were selected for POC coverage until 15% of national viral load volumes were covered* Only primary healthcare facilities and district level hospitals were included.
4. All POC	A complete switch from centralized to POC testing	All facilities in South Africa that currently send viral load specimens to centralized laboratories were allocated POC instruments according to their viral load volumes.

⁎ 15% coverage is typically required to ensure the lowest cost per test in pricing agreements with volume commitments.

The facility selection criteria are outlined in Table 1. All facilities that currently send viral load specimens for testing at centralized laboratories were included in Strategy 1 (“Status quo”) and 4 (“All POC”). For the other two strategies, we included health facilities that would be considered suitable candidates for POC placement within the context of the HIV program: clinics, community health centers and district hospitals that provide out-patient services to HIV patients and that do not generally have onsite laboratories. Strategy 2 targeted only facilities that reported low viral suppression rates and/or high numbers of unsuppressed viral loads (“Unsuppressed targeted”). For strategy 3, POC instruments were allocated to facilities that performed the worst on three measures, (1) low viral suppression, (2) long TAT, 3) and high specimen rejection rates, until 15% of viral load testing volumes were covered (“Combination targeted”). In addition, in order to minimize costs, targeting involved identifying candidate facilities that had sufficient viral load volumes (approximately 4–7 tests per POC device per day) such that instrument capacity would be maximized.

### 2.1 Capacity and allocation of POC instruments

We assumed the Abbott m-PIMA™ could perform between 4 and 7 viral load tests per day with each test taking less than 70 min to conduct [12]. The testing capacity for the Cepheid Xpert was determined assuming a 7-h day, 90 min per test multiplied by the number of modules (GeneXpert II, IV, XVI) [13]. For the POC strategies, POC instruments were allocated to facilities and matched as closely as possible to the viral load volumes at the facility and the capacity of the POC instruments. In strategy 3, new facilities were allocated POC instruments until 15% of national viral load volumes were covered; strategy 4 involved deploying instruments at facilities until each facility's viral load volume was met by POC capacity. Strategies 3 and 4 had mixed-technology POC strategies, whereby either m-PIMA and/or Xpert POC instruments were allocated depending on the volume of viral loads required at the facility. Anecdotal evidence from NHLS suggested that at low volumes, the m-PIMA was easier to operate by lay staff in health facilities. For strategy 3 and 4, m-PIMA was first allocated to facilities with less than 7 expected daily viral loads, and then Xperts were allocated and their capacity matched to the daily viral load volumes at the selected facilities Strategy 2 differed slightly from the other two POC strategies in that only patients suspected of being unsuppressed (measured using the number of expected unsuppressed viral loads) were tested using POC. Any additional instrument capacity was used to test suspected suppressed viral loads and excess viral loads were sent to the centralized laboratory for testing. Differences in the total POC tests conducted by mPIMA or Xpert sub-strategies is driven by differences in the POC instrument capacity. In strategy 2, given the smaller POC volumes based on the number of expected unsuppressed viral loads a day, a mixed technology POC strategy was not considered.

### 2.2 Cost analysis

For centralized viral load testing, we used the NHLS ‘charge’ price (for which we had permission to use) from 2017 (inflated to 2019). The price is all-inclusive of consumables, specimen transport, testing and result delivery. The m-PIMA cost is based on estimates from the NHLS with the upper-bound based on an outright instrument purchase arrangement with low test volume commitment and the lower-bound on an instrument lease arrangement with the largest volume commitment [14]. Building on the approach used by Simeon et al., Cepheid Xpert viral load test costs were based on instrument procurement and reagent prices as reported by the Global Fund and the range reflects low to high volume commitments for reagent prices as well as low to high instrument capacity utilization [15]. Both the costs for m-PIMA and Xpert included other costs sourced from NHLS: staff costs associated with running samples, interpreting and recording results; the consumables used per test; the training of the staff nurse; a laboratory manager's oversight time; the cost of a POC coordinator that oversees the administrative costs of running a POC program at scale, support equipment (e.g. micro centrifuge), support travel and external quality assessment [14]. All costs are reported in 2019 US dollars (Table 2).

**Table 2:** Viral load testing costs*.

Technology	Cost per viral load test		Sources
	USD	USD (range)	
Abbott m-PIMA™	37·68	29·51–45·85	NHLS costing[14]
Cepheid Xpert II	28·33	25·11–43·16	Global Fund procurement [15] and NHLS costing
IV	25·40	23·35–34·37	
XVI	23·98	22·50–30·11	
Centralized testing	24·46		NHLS price list

⁎ Costs reported in 2019 USD using average monthly exchange rate for 2019. www.resbank.co.za.

### 2.3 Cost-effectiveness and budget impact analysis

For each strategy and POC technology, we determined the total cost, effectiveness (defined as the total expected number of people with suppressed viral load (<1000 copies/ml)) and incremental cost-effectiveness ratio (ICER) based on an expected improvement in suppression rates from POC adoption ($ amount per additional person suppressed). A cost-effectiveness frontier was created to determine the strategies that are more likely to be considered cost-effective at different levels of budget availability.

The expected improvement in suppression from POC adoption were based on the results from the South African-based Simplifying HIV TREAtment and Monitoring (STREAM) POC viral load trial[6]. Using a suppression threshold of <1000 copies/ml, the proportion of patients virally suppressed was expected to improve by 10·8% for POC viral load tests compared to centralized testing, and by 62% for those previously unsuppressed (the percent change between POC and standard of care for those who were unsuppressed at baseline and who became suppressed, using the threshold of 1000 copies/ml) (Strategy 2). Current levels of viral suppression were adjusted by these factors and multiplied by the number of expected viral load tests to determine the new number of expected people with suppressed viral loads.

The national total cost for each strategy was calculated by estimating the viral load volumes to be tested on each platform (centralized, Xpert and m-PIMA). The average utilization of the platform given its capacity was calculated to determine the associated cost of the viral load test that corresponded to the instrument utilization: lowest costs for the highest utilization and high costs for the lowest utilization. Costs were reported for a 1-year time period and included financial costs from the provider's perspective. All costs included in the analysis are outlined above and reported in Table 2. Additional economic costs and the health and cost impact of different strategies on HIV transmission were not included in this analysis.

### 2.4 Sensitivity analysis

To assess the robustness of our model and conclusions, we conducted a multiple one-way sensitivity analysis varying the cost per POC viral load test (reducing the cost of m-PIMA to the costs determined for Xpert II and reducing Xpert reagent costs to ensure cost-neutrality with the status-quo) and varying the effectiveness of POC viral load testing in improving viral suppression (+/−50% in the absence of additional trial evidence) and assuming a less than perfect ability of the clinic to target those who are unsuppressed (from 100% to 10% which is conservative given that past viremia is predictive of future viremia [4]). Changing the ability to target a patient with viremia does not change the total number of tests being conducted on POC. It rather assumes that clinicians are only able to target one out of every ten patients who are unsuppressed for POC testing, and that the remaining nine tests are done on patients that do not have viremia if there is sufficient POC capacity (or else referred for centralized testing). This changes the potential effectiveness of POC as the effectiveness of POC is more pronounced if the patient is initially unsuppressed. To assess the interaction of these uncertain parameters, we additionally conducted a probabilistic sensitivity analysis through 1000 Monte Carlo simulations. For each parameter in the one-way sensitivity analysis (excluding the threshold analysis on the Xpert reagent costs), we sampled from a uniform distribution between the high and low value.

### 2.5 Ethics

The National Priority Programmes of NHLS currently holds blanket ethics approval (HREC M160978) for retrospective analyses of laboratory data through the Integrated Laboratory Data Analysis for Care (ILDAC) program. This blanket approval covers projects that make use of existing, routinely collected laboratory data relevant to the national control programs. Only aggregate facility-level count data were used for this analysis.

### 2.6 Role of the funding source

This work was supported by the Bill & Melinda Gates Foundation through the Innovation in Laboratory Engineered Accelerated Diagnostics investment (grant number OPP1171455). The funders had no role in the study design, data collection and analysis, decision to publish, or preparation of the manuscript. The authors’ views expressed in this publication do not necessarily reflect the views of the Bill & Melinda Gates Foundation.

## 3 Results

We included 4216 healthcare facilities in the analysis that send over 5.1 million specimens for viral load testing at centralized laboratories each year. Most facilities (63%, *N* = 2655) have less than four specimens per day that need to undergo viral load testing and contribute a fifth of annual viral load volumes. Just 8% (*N* = 322) of facilities contribute over a third of annual viral load volumes and send on average more than 14 viral load specimens to centralized laboratories for testing each day (Table 3).

**Table 3:** Distribution of facilities and viral load volumes.

Viral load daily test volume category	Number of facilities	% facilities	Average viral load tests per year	Annual viral load tests per year	% volumes
Less than 1 per day	1088	26%	116	126,647	3%
Between 1 and 4 per day	1567	37%	558	874,548	17%
Between 4 and 7 per day	634	15%	1329	842,340	16%
Between 7 and 14 per day	605	14%	2390	1,446,078	28%
Greater than 14 per day	322	8%	5753	1,852,577	36%
**Total**	**4216**	**100%**	**1220**	**5,142,190**	**100%**

Table 4 presents descriptive statistics for facilities allocated to each strategy at baseline, prior to POC adoption. Both strategy 1 and 4 covered all 4216 facilities in the analysis. At baseline in these facilities the mean daily viral load volumes per facility for the full sample was 4·9 with a mean number of 0·7 unsuppressed viral loads expected per day per facility (Table 4). The mean specimen rejection rate was 5% and the mean TAT was 60 h. Strategy 2 targeted higher volume facilities, specifically targeting facilities that expect to test more than 1 unsuppressed viral load/day – a mean of 1·9 per facility per day. Lastly, strategy 3, which targeted low-performing facilities, had, at baseline, lower viral load volumes (3·3 per facility per day), higher TAT (87 h), higher specimen rejection rates (8%) and higher unsuppressed rates (26% versus 17%).

**Table 4:** Facility characteristics at baseline for each strategy.

Strategies	Number of facilities		Mean daily viral load volumes (SD)	Mean daily unsuppressed viral loads (SD)	Mean rejection rate (SD)	Mean TAT (SD), h
	Centralized	POC				
1. Status quo	4216	0	4·9 (7·0)	0·7 (1·1)	5% (6%)	60 (39)
2. Unsuppressed targeted	3353	863	13·0 (9·1)	1·9 (1·0)	5% (3%)	53 (38)
3. Combination targeted	3260	956	3·3 (4·3)	0·7 (0·9)	8% (8%)	87 (50)
4. All POC	0	4216	4·9 (7·0)	0·7 (1·1)	5% (6%)	60 (39)

**Fig. 1:**
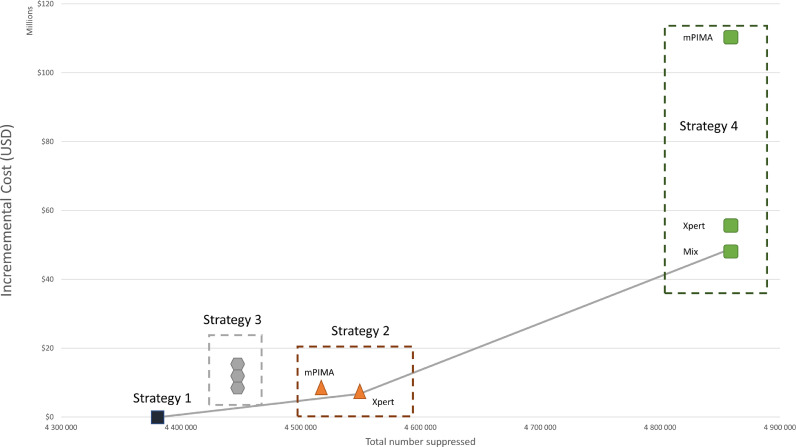
Cost-effectiveness frontier. All strategies above the cost-effectiveness frontier (the gray line) are considered either more costly and less effective, or more costly and less cost-effective.

The centralized network (strategy 1) costs $126 million annually with viral load suppression of 85·2% (Table 5). Strategy 2 (targeted testing) using the Xpert increased viral suppression by 3·3 percentage points and was considered highly cost-effective at $40 per additional person suppressed compared to the centralized network, requiring an additional $6·7 million annually. Should resources allow, the all-POC strategy using a mix of Xpert and m-PIMA may be cost-effective with an ICER of $136 per additional person suppressed compared to the next most cost-effective strategy, Strategy 2. The all-POC strategy would require an additional $48·7 million annually compared to the centralized network, with viral load suppression of 94.5%. As shown by the cost-effectiveness frontier (Fig. 1), all other strategies were found to be either more costly and less effective or more costly and less cost-effective (i.e. dominated) in the incremental analysis.

**Table 5:** Health and economic outcomes of point of care adoption strategies.

Strategy	Description	Technology	% Viral load volumes tested by platform			Total cost (2019 USD)	Total number suppressed (%)	ICER ($/additional person suppressed)
			*m-PIMA*	Xpert	Centralized			
1	Status-quo	Centralized	0%	0%	100%	$125,802,848	4,382,475 (85·2%)	-
2	Unsuppressed targeted*	Xpert only	0%	34%	66%	$132,516,289	4,548,682 (88·5%)	$40
2	Unsuppressed targeted *	m-PIMA only	27%	0%	73%	$132,864,944	4,517,130 (87·8%)	dominated
3	Combination targeted⁎⁎	Xpert only	0%	15%	85%	$136,218,447	4,447,944 (86·5%)	dominated
3	Combination targeted⁎⁎	mix	13%	2%	85%	$139,751,552	4,447,944 (86·5%)	dominated
3	Combination targeted⁎⁎	m-PIMA only	15%	0%	85%	$142,317,674	4,400,744 (86·5%)	dominated
4	All POC	mix	36%	64%	0%	$174,505,308	4,857,683 (94·5%)	$136
4	All POC	Xpert only	0%	100%	0%	$180,661,135	4,857,683 (94·5%)	dominated
4	All POC	m-PIMA only	100%	0%	0%	$235,769,172	4,857,683 (94·5%)	dominated

⁎ Targeted to facilities with highest number of virally unsuppressed patients.

⁎⁎ Targeted to facilities that have a combination of low viral suppression, long turnaround time and high specimen rejection rates.

### 3.1 Sensitivity analyses

When assuming POC viral load resulted in either lower/higher levels of viral suppression than baseline (reduction of 50% or an improvement of 50%), ICERs proportionally increased/decreased from $40 to $81/$27 for strategy 2 (Xpert-only) and from $136 to $588/$77 per additional person suppressed for strategy 4 (technology mix). Assuming less than perfect targeting in strategy 2 (only one out of every ten unsuppressed patients will be targeted for POC testing) increased the ICER marginally from $40 to $42 (Table 6). Neither of these changed the results regarding the optimal strategy for implementation.

**Table 6:** Sensitivity analysis – imperfect targeting strategy 2 (100–10%).

Strategy	Description	Technology	Total Cost (2019 USD)	Total number suppressed (%)	ICER ($/additional person suppressed)
1	Status-quo	Centralized	$125 802 848	4 382 475 (85.2%)	$0
2	Unsuppressed targeted	Xpert only	$132 516 289	4 548 682 (88.5%)	$40
2*	Unsuppressed targeted	Xpert only	$132 516 289	4 541 993 (88.3%)	$42

⁎ Sensitivity analysis on strategy 2.

Assuming a lower cost per viral load test on m-PIMA using baseline cost of a viral load test on Xpert II (cost reduction of 6–33% depending on utilization), from a current cost of $37·68 ($29·51–$45·85) to a new cost of $28·33 ($25·11–$43·16), changed the order of the cost-effectiveness of the POC adoption strategies for strategy 2. Strategy 2 remained the least costly after the centralized strategy, but the m-PIMA sub-strategy was added to the cost-effectiveness frontier with an ICER of $7 per additional person suppressed. The order of the remaining strategies remained the same, but the strategies that used m-PIMA technology became less costly. A reduction in the Xpert reagent cost of 30% (from $14.75 to $11.40) rendered strategy 2 cost-neutral with the status-quo.

**Fig. 2:**
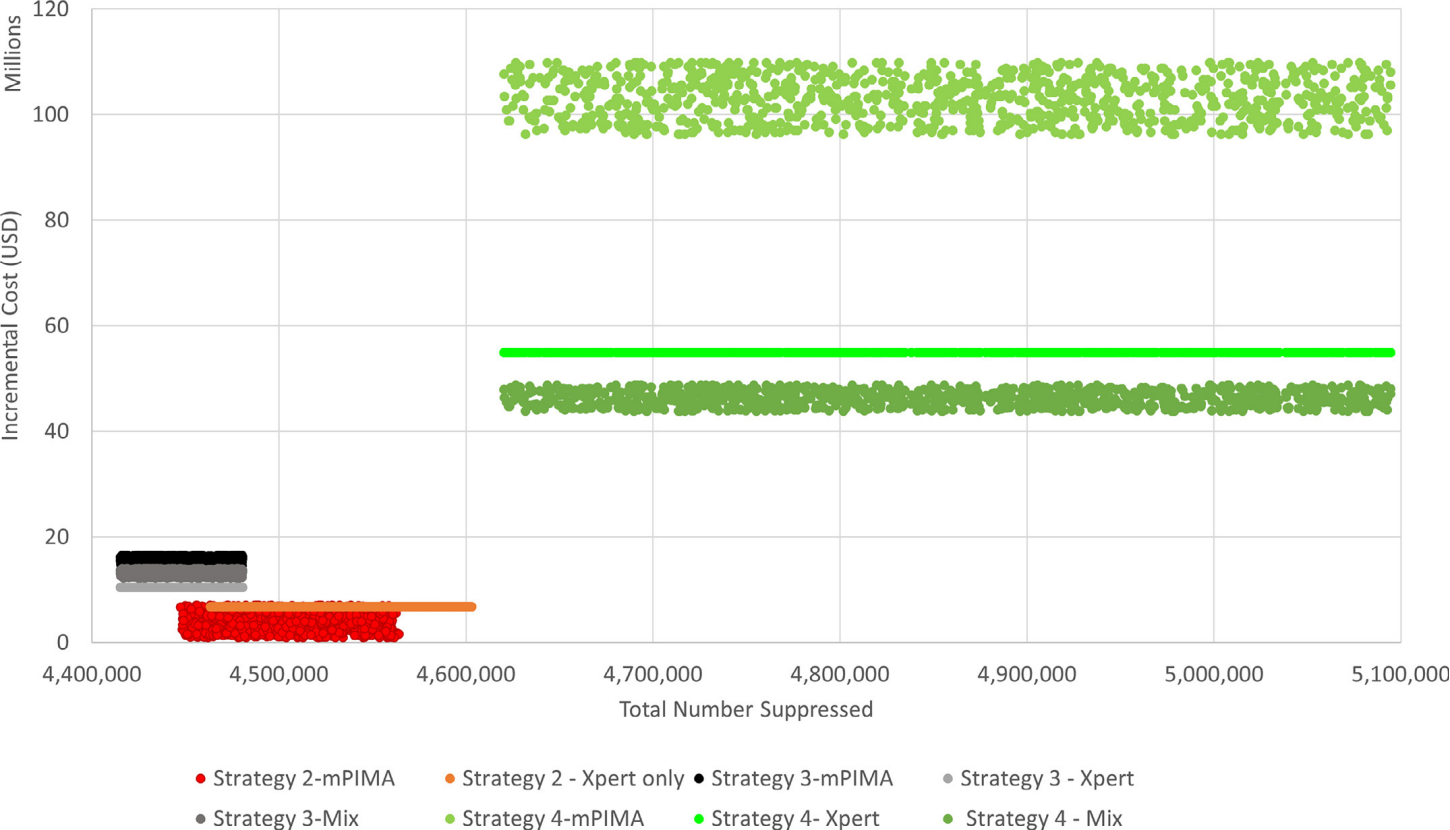
Probabilistic Sensitivity Analysis – Cost-effectiveness scatter plot. Strategies with m-PIMA technology drives variation in incremental costs in simulation output. Variation in the effectiveness (number of persons suppressed) is greater for the full POC strategy (strategy 4) versus the partial POC strategies (strategy 2).

Strategy 2 (Xpert-only) and Strategy 4 (technology mix) remained cost-effective in the probabilistic sensitivity analysis of the interaction of uncertainty around m-PIMA costs, POC impact on viral suppression, and ability to target viremic patients. However, the order of the cost-effectiveness of the POC adoption strategies for strategy 2 changed – the m-PIMA sub-strategy was less costly for a given level of viral suppression (Fig. 2). All other strategies were dominated in the incremental analysis.

## 4 Discussion

Our model-based analysis evaluated the cost and health impact of implementing various strategies of POC VL testing in South Africa. Given clinical benefits from improved viral suppression rates due to POC viral load testing, we find that the most cost-effective strategy for viral load POC adoption in South Africa is likely a targeted approach, with POC instruments placed at larger facilities with high numbers of patients experiencing viral failure. This is projected to cost $40 per additional person suppressed compared to the centralized network. Should resources allow, the all-POC strategy using a mix of Xpert and m-PIMA may be cost-effective as well. However, both strategies require an increase in the health expenditure budget, 5·3% for Strategy 2 and 38·7% for Strategy 4, which may impact their affordability. A reduction in reagent prices for the POC tests would improve affordability at scale. Importantly, should the reagent cost of Xpert be reduced by 30%, from $14.75 to $11.40, Strategy 2 would be cost-neutral with the status quo. These findings are particularly important for countries that have already scaled-up viral load programs, such as South Africa, or have a strong, comprehensive well-functioning viral load testing network. Critically, for any of these strategies to be considered cost-effective, patient-level benefit of POC viral load testing in terms of improved retention or viral suppression must be observed in routine practice. Reductions in retention/suppression improvements would result in a higher cost per additional person suppressed, but the annual viral load budget estimates would remain the same.

The results demonstrate that instrument utilization matters: higher volume facilities have better instrument utilization and consequently lower POC costs. The most cost-effective strategy (strategy 2) was the use of targeted POC at facilities nationally that expect more than one unsuppressed viral load a day and where additional instrument capacity is used to conduct viral load tests on virally suppressed patients. The cost-effectiveness of strategy 2 is largely driven by the higher volumes at these facilities which allow for improved instrument utilization and consequently lower costs per viral load test. This result might not be acceptable to health policy makers who are seeking to ensure equity through extending access to smaller clinics, or to improve access to patients at low volume clinics with long TAT and high specimen rejection rates. Sufficient budget for viral load testing would have to be available to achieve this particular goal.

A number of studies have assessed POC diagnostic accuracy of [16], [17], [18], [19], [20], [21], costs [9], cost-effectiveness [10] and clinical outcomes [6,8,22] relative to centralized testing, but to our knowledge, this is the first paper that has assessed the feasibility of different adoption strategies for POC viral load monitoring on a national scale. This analysis is also novel in that it incorporates both costs and clinical outcomes and uses facility-level data to match viral load demand to equipment capacity in order to minimize costs.

There are several limitations to this analysis. Firstly, there may be benefits of POC viral load testing that extend beyond what is modelled in the present analysis. POC testing allows for more patient-centered care as patients can receive a blood draw for a viral load test, even after the transport for the centralized testing has left for the day [23]. As such, viral load testing access and consequently volumes may increase with the availability of POC viral load testing, this is particularly important in countries that have yet to achieve national viral load coverage. Point-of-care results may also allow faster referral into more efficient, decentralized models of differentiated ART delivery[24] and, by improving specimen rejection rates, decrease the time to a second viral load after rejection and decrease the amount of unsuppressed time in the population.

Secondly, we did not consider the polyvalent nature of the instruments. Additional capacity on the instruments could be used to conduct other tests – early infant diagnosis, TB diagnosis, sexually transmitted infections diagnosis, among others. This would further decrease costs in the POC strategies. Thirdly, we used a threshold of 1000 copies/ml to determine the benefits of POC in increasing the number of people on ART who are classified as suppressed. This approach however does not take into account the South African guidelines on viral load failure thresholds nor to the limit of detection of the different platforms. South Africa has recently adjusted the viral load threshold for detecting viral failure to >50 copies/ml from 400 copies/ml [25], below the WHO-recommended 1000 copies/ml threshold. Whilst we used the WHO threshold to ensure greater comparability with other low resource settings, the South African program might need to additionally consider the cost of misclassification of m-PIMA (with a limit of detection of 314 copies/ml) versus Xpert (limit of detection of 38 copies/ml) [26,27]. Fourth, we do not take into account the capacity and ability of certain facilities to adopt POC testing and incorporate it into clinic operations [28] and we have assumed that nurses will be able to perform plasma sample processing and POC testing in routine healthcare facilities [29]. A limitation of the low performing facility strategy is that low performing facilities might be dysfunctional in ways that will affect the effective operationalization of POC. Fifth, we have only evaluated the one-year impact on the national viral load testing budget. While several POC interventions did not prove to be cost saving in the short term, they could be cost-effective or possibly cost saving if prevention of HIV transmission was considered. Lastly, there may be other, less costly methods to return the results directly to a patient and possibly confer the same patient-level benefit as observed in the STREAM POC viral load trial, such as text result-delivery directly to the patient [30].

Assuming POC viral load testing improves patient viral suppression, the most cost-effective strategy for viral load POC adoption in South Africa is likely a targeted approach, in which POC are placed in larger facilities with high rates of viral failure. The results of this analysis are generalizable across a broad number of countries, in particular countries that have already scaled up their centralized laboratory systems and are looking to improve access for the harder to reach facilities and/or improve outcomes. This methodology can also be adapted to improve laboratory access for other conditions than HIV, achieve optimal placement of equipment, and evaluate trade-offs between centralized and decentralized laboratory testing.

## Authors’ contributions

BEN, WS and SJG conceived the study. SJG, BEN, WS, TC, JD, MS, PD, JD, NG acquired and analyzed data for the model. SJG and BEN developed the model. SJG and BEN interpreted model results. SJG and BEN wrote the first draft of the manuscript. SJG, BEN, WS, TC, JD, MS, PD, JD, NG read and approved the final manuscript.

## Declaration of Competing Interest

PKD reports receiving consulting or speaking fees from Gilead Science, and research support from the NIH, CDC, Gilead Sciences, and the Bill and Melinda Gates Foundation. He declares that he has no competing interests. MS reports grants from National Institutes of Health outside the submitted work. SJG, TC, WS, NG, BEN, and JD have nothing to disclose.

## Appendix Supplementary materials

Image, application 1
